# TFIIS enhancement of Pol II cleavage is under kinetic control

**DOI:** 10.1093/nar/gkag874

**Published:** 2026-09-16

**Authors:** Ryan M Requijo, Nasib Karl Maluf, David A Schneider, Aaron L Lucius

**Affiliations:** Department of Chemistry, University of Alabama at Birmingham, Birmingham, AL 35294, United States; Department of Chemistry, University of Alabama at Birmingham, Birmingham, AL 35294, United States; Department of Biochemistry and Molecular Genetics, University of Alabama at Birmingham, Birmingham, AL 35294, United States; Department of Chemistry, University of Alabama at Birmingham, Birmingham, AL 35294, United States

## Abstract

The eukaryotic RNA polymerases (Pols) contain subunits or interact with *trans*-acting factors that confer robust endonuclease activity. A12.2 and C11 are subunits of Pol I and Pol III, respectively. Pol II, however, recruits transcription factor IIS (TFIIS). This evolutionary divergence of A12.2 and C11 being bona fide subunits while TFIIS is a *trans*-acting factor suggests functional divergence of these domains. To uncover the mechanistic impact of TFIIS on Pol II-catalyzed nucleotide incorporation and endonuclease activity, single turnover *in vitro* transcription assays were performed with *Saccharomyces cerevisiae* TFIIS and Pol II. Nucleotide incorporation time courses were collected as a function of both TFIIS and nucleotide concentrations. Global nonlinear least-squares analysis of these time courses revealed that TFIIS binds to elongation complexes after nucleotide incorporation because TFIIS binding is slow relative to correct nucleotide incorporation. However, if subsequent nucleotide incorporation is slow, TFIIS has adequate time to bind and activate Pol II’s endonuclease activity. From these findings, we hypothesize that the mechanism of TFIIS-stimulated endonuclease activity is kinetically controlled.

## Graphical Abstract

**Graphical Abstract:**
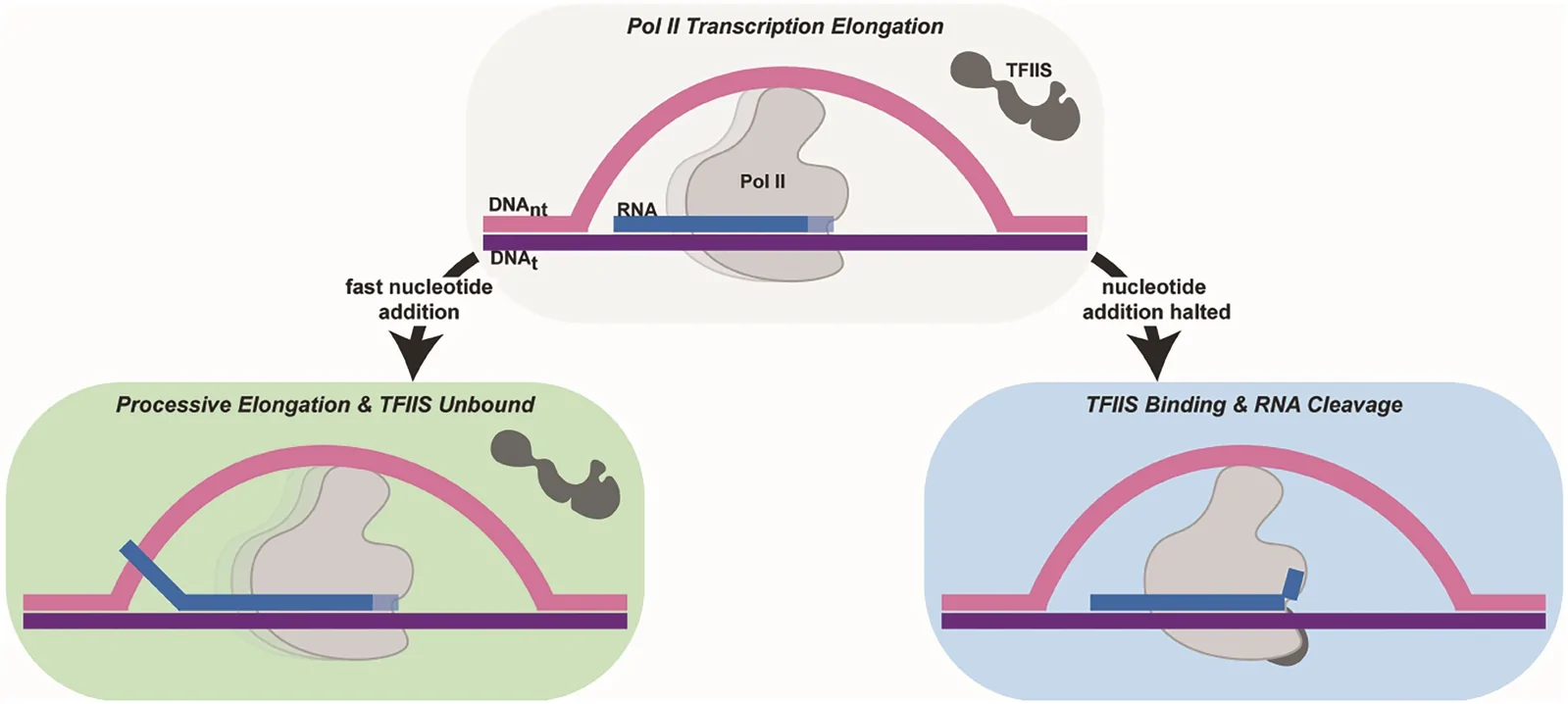

## Introduction

Archaea and bacteria express a single RNA polymerase (RNAP) responsible for synthesizing all RNA in these domains of life [1–4]. Eukaryotes, however, have evolved to express at least three DNA-dependent RNA polymerases (Pols) with specialized roles in transcription [2, 5, 6]. Pol I synthesizes ribosomal RNA (rRNA), Pol II primarily synthesizes messenger RNA (mRNA), and Pol III primarily synthesizes transfer RNA (tRNA). The evolution of specialist Pols in eukaryotes implies functional divergence must have also emerged.

It is hypothesized that Pol II evolved from archaeal RNAP while Pol I and Pol III evolved from Pol II through the incorporation of transcription factors as intrinsic subunits [7–9]. This structural divergence of the Pols has been well defined [2, 5, 10–15], but mechanistic differences that may have arisen are not fully understood. Using transient-state kinetics, we have put significant effort into defining the mechanistic differences between the three Pols [16–31].

The eukaryotic Pols are multi-subunit enzymes with Pol I, Pol II, and Pol III consisting of 14, 12, and 17 subunits, respectively [2, 14]. Of these subunits, five are shared: Rpb5, Rpb6, Rpb8, Rpb10, and Rpb12. Rpb5 and Rpb6 are core catalytic subunits conserved between the three Pols while the remaining core catalytic subunits differ but are homologous to one another [14], On the other hand, subunits peripheral to the core have diverged with Pol I, Pol II, and Pol III having four, two, and seven peripheral subunits, respectively [2, 5, 14]. Pol II, having the lowest number of subunits, recruits transcription factors to perform the functions of their homologous domains in Pols I and III, and this further suggests that Pols I and III evolved from Pol II through the incorporation of transcription factors as intrinsic subunits [2, 7, 9, 14].

A12.2 and C11 are intrinsic subunits of Pol I and Pol III, respectively. These subunits confer endonuclease activity to the Pols, a function required for recovery of stalled transcription elongation complexes (ECs) through cleavage of a phosphodiester bond within the nascent RNA chain [8, 18, 32, 33]. In contrast to Pols I and III, transcription factor IIS (TFIIS) is a trans-acting factor that must be recruited to Pol II to confer endonuclease activity [7, 8, 34, 35]. While it is clear these subunits have diverged, the impact of this divergence on the elementary reaction mechanism is not well understood.

We have developed a single turnover transient-state kinetics approach that has allowed us to report elementary mechanisms of transcription elongation catalyzed by Pol I, Pol II, Pol III, and Pol I lacking A12.2 (ΔA12 Pol I) [16, 18, 22, 25–27, 30]. For a single nucleotide incorporation catalyzed by Pol I, a fast and irreversible bond formation step (∼200 s^-1^) followed by a cleavage step (∼0.4 s^-1^) are observed [16]. In contrast, for ΔA12 Pol I, bond formation is reversible and slower (∼60 s^-1^) while the cleavage step is no longer detected [18]. Similarly, Pol II exhibits reversible and slower (∼80 s^-1^) bond formation in the absence of its cleavage-conferring transcription factor, TFIIS [27]. From these observations, we hypothesized that A12.2 is involved in each nucleotide addition reaction, and the removal of A12.2 makes Pol I more “Pol II like.”

We observed a similar phenomenon in the context of multiple nucleotide incorporations. For nine successive nucleotide incorporations, Pol I was best described by a mechanism with irreversible steps between each nucleotide incorporation [22]. On the other hand, ΔA12 Pol I and Pol II show dramatically different kinetics, requiring reversible steps between each nucleotide addition [22, 26, 30]. Again, removal of A12.2 from Pol I resulted in a mechanism that was similar to Pol II.

Pol I’s mechanism of transcription elongation being more like Pol II upon the removal of A12.2 supports the evolutionary hypothesis that Pol I evolved from Pol II by incorporating transcription factors as *bona fide* subunits. Moreover, this provides some insight into the hypothesis that Pol I incorporated TFIIS to generate A12.2. However, the converse of this result has not been demonstrated. In other words, does the addition of TFIIS to Pol II make its mechanism of nucleotide incorporation and cleavage more “Pol I like”?

Using transient-state kinetic techniques, we tested the impact of TFIIS on the single and multi-nucleotide incorporation reactions catalyzed by Pol II. Here, we show that TFIIS binds after nucleotide addition to two transient conformations of the Pol II EC. By comparing both single- and multi-nucleotide addition experiments, all evidence suggests that TFIIS does not remain bound to the EC through multiple nucleotide additions. In fact, TFIIS only binds after nucleotide addition when the next cognate nucleotide is not present. This contrasts with the Pol I A12.2 subunit which is present in the EC throughout the transcription cycle. We hypothesize that, evolutionarily, the TFIIS subunit did not get incorporated into Pol II as a bona fide subunit because Pol II-catalyzed nucleotide addition is on a comparable time scale as TFIIS binding and cleavage. Thus, cleavage occurs through kinetic competition between nucleotide addition to a stalled EC and binding of TFIIS to promote cleavage. Therefore, there was no evolutionary pressure to incorporate the subunit.

## Materials and methods

### Buffers

Buffers and salt solutions were vacuum filtered using 0.2 µm pore size and 75 mm diameter Fisherbrand^™^ Disposable PES Bottle Top Filters (Fisher Scientific) unless otherwise stated.

The reaction buffer for manual mixing and quench-flow experiments is composed of the following: 40 mM KCl, 20 mM Tris-acetate (OAc) pH 8.23 at 25°C, 2 mM dithiothreitol (DTT), and 0.2 mg ml^-1^ bovine serum albumin (BSA). For EC stability experiments, [KCl] was increased to 750 mM with all other buffer components and concentrations remaining the same.

### Proteins

Pol II was endogenously expressed in and purified from *Saccharomyces cerevisiae* as previously published [36].

To overexpress recombinant *S. cerevisiae* TFIIS, *Escherichia coli* Rosetta (DE3) cells (Novagen) were transformed with pRSFDuet vector (Novagen) containing the TFIIS gene fused with an N-terminal His6-tag via a linker containing a TEV protease site. The plasmid was received from Kenji Murakami, and their previously published protocol was adapted to overexpress and purify TFIIS [37]. Briefly, the transformed cells were grown in 2xYT at 30°C and induced at OD_600_ = 0.5 with 0.2 mM isopropyl β-D-1-thiogalactopyranoside (IPTG) for 3 h. Cells were harvested then lysed using a French press cell disruptor (Avestin), and the soluble fraction of the cell lysate was purified using HisPur Ni-NTA resin (Fisher Scientific). After elution of TFIIS from the Ni-NTA resin, the His6-tag was cleaved by incubating the eluate with TEV protease for 24 h at 4°C. Further purification of TFIIS was accomplished via Ni-NTA (Cytiva), heparin (Cytiva), and SP Sepharose (Cytiva).

### Nucleotides, nucleic acids, heparin, and BSA

Nucleotides, nucleic acids, heparin, and BSA were prepared as previously published [36]. Sequences of the RNA primer, template DNA, and non-template DNA are as follows:

RNA primer: 5′-AUCGAGAGG-3′
Template DNA: 5′-ACCAGCAGGCCGATTGGGAT GGGTATTCCCTCCTGCCTCT CGATGGCTGTAA GTATCCTAT AGG-3′
Non-template DNA: 5′-CCTATAGGATACTTACAGCCAT CGAGAGGCAGGAGGG AATACCCATCCCAATC GGCCTGCTGGT-3′

### Radiolabeled elongation complex assembly

**Figure 1.**
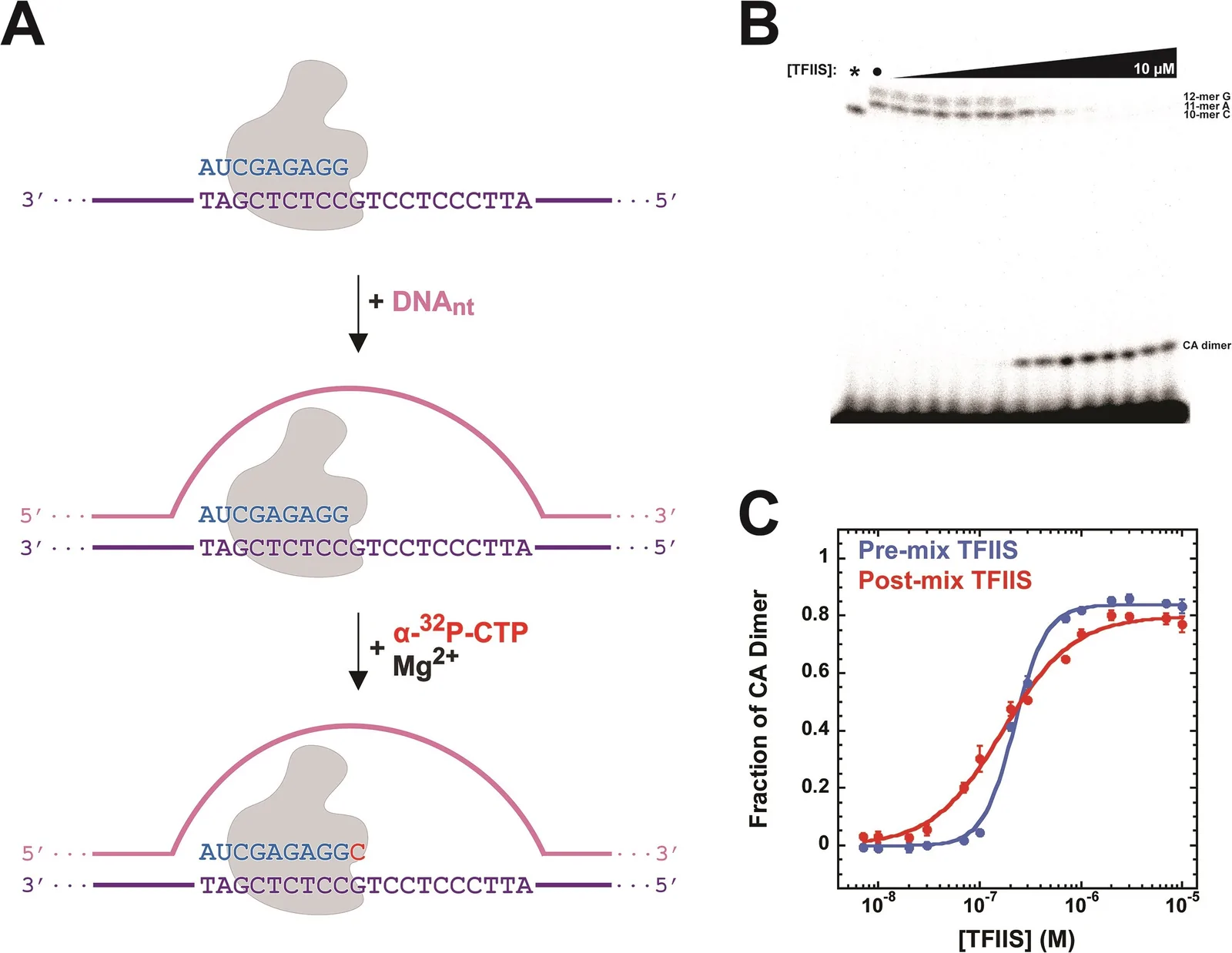
EC formation and TFIIS manual mixing. (**A**) *In vitro* EC assembly schematic. EC solution was prepared by incubating Pol II (light gray) with a pre-annealed 9-mer RNA primer (cyan) and DNA template strand (purple). Relevant portions of the sequences are shown. The DNA nontemplate strand (pink) was then added to generate a 9-mer EC. α-^32^P-CTP (red) and Mg^2+^ were added to the 9-mer EC to form radiolabeled 10-mer EC. EDTA was added to chelate Mg^2+^ and halt the radiolabeling reaction. (**B**) Representative gel of TFIIS manual mixing. To determine the concentration of TFIIS needed to saturate Pol II endonuclease activity, EC solution was manually mixed with a solution containing ATP, Mg^2+^, and heparin. Prior to mixing, TFIIS was either pre-incubated with the EC solution or with the ATP solution. In both cases, the post-mixing concentration of TFIIS varied from 7 nM to 10 µM. All reactions were quenched with 1 M HCl after 60 s. The representative gel is from an experiment where TFIIS was pre-incubated with the EC solution. Each row of the denaturing gel represents a different intermediate or product while each column represents a different concentration of TFIIS. The * indicates a reaction without TFIIS/ATP while the • indicates a reaction without TFIIS. (**C**) TFIIS activity isotherm. To visualize TFIIS enhancement of endonuclease activity, the fraction of CA dimer was plotted as a function of TFIIS concentration. TFIIS pre-incubated with EC (i.e. pre-mix TFIIS, blue) and TFIIS pre-incubated with ATP (i.e. post-mix TFIIS, red) were both described by a cooperative model (see Eq. 1 and Table 1).

EC solutions were prepared as previously published [36]. A schematic representation of the assembly of the EC and radiolabeling of a 9-mer RNA catalyzed by Pol II is given in Fig. 1A. First, the 9-mer RNA is annealed to the template DNA (DNA_t_) before incubation with Pol II. During this incubation, Pol II is allowed to bind to this RNA–DNA hybrid for 20 min on ice followed by 20 min at ambient temperature. The nontemplate DNA (DNA_nt_) is then added and allowed to incubate for 40 min at ambient temperature to form the EC. This is followed by addition of α-^32^P-CTP and Mg^2+^, which allows for bound Pol II to catalyze incorporation of ^32^P-CMP at the 3′ end of the RNA. After 10 min, this reaction is halted with the addition of EDTA to chelate Mg^2+^. Thus, EC solutions prepared in this way contain Pol II bound to a radiolabeled 10-mer RNA, which serves as the starting point for subsequent reactions.

The initial concentrations of the components of the EC solution are as follows: ∼80 nM Pol II, 162.75 nM RNA, 54.26 nM template DNA, 162.75 nM nontemplate DNA, ∼5 nM α-^32^P-CTP, 100 µM Mg(OAc)_2_, and 1.1 mM EDTA-K_3_. These concentrations are diluted 2-fold after 1:1 mixing.

### Manual mixing experiments

To perform manual mixing experiments, EC solution containing radiolabeled Pol II ECs was prepared as previously described. Another solution containing ATP was also prepared. The initial concentrations of the components of the ATP solution are as follows: 2 mM ATP, 18 mM Mg(OAc)_2_, and 0.054 mg ml^-1^ heparin.

TFIIS was added to one of the solutions and allowed to incubate for 10 min. In “pre-mix” experiments, TFIIS was added to the EC solution, which allows for binding of TFIIS with Pol II ECs before initiation of any subsequent reactions. In “post-mix” experiments, TFIIS was added to the ATP solution, which allows for TFIIS binding and initiation of subsequent reactions to happen simultaneously. For both pre- and post-mix experiments, the initial concentration of TFIIS was varied from 14 nM to 20 µM.

The EC and ATP solutions were mixed 1:1 via pipetting to initiate the nucleotide incorporation and cleavage reactions. Thus, the concentrations of both solutions were diluted 2-fold, and the final TFIIS concentration ranged from 7 nM to 10 µM. Each reaction proceeded for 60 s at 25°C before quenching with 1 M HCl. Gel electrophoresis and quantification of the products of these reactions were carried out as previously published [36].

### Quench-flow time courses

To investigate the kinetics of Pol II-catalyzed nucleotide incorporation and cleavage in the presence of TFIIS, time courses were collected at 25°C using a chemical quench-flow (KinTek). In a quench-flow, the contents of two syringes are rapidly mixed within 2 ms, and the contents of the two syringes are diluted 1:1. Reaction time, Δt, was varied from 5 ms to 60 s before each reaction was rapidly quenched with 1 M HCl.

For single nucleotide incorporation time courses, two syringes were prepared. The EC syringe contained ∼80 nM Pol II, 162.75 nM RNA, 54.26 nM template DNA, 162.75 nM non-template DNA, ∼5 nM α-^32^P-CTP, 100 µM Mg(OAc)_2_, 1.1 mM EDTA-K_3_, and varying concentrations of TFIIS. The ATP syringe contained varying concentrations of ATP, 18 mM Mg(OAc)_2_, and 0.054 mg ml^-1^ heparin. Note that the concentrations reported in the text correspond to those achieved after rapid mixing of these two syringes and consequently are reduced by a factor of 2.

For multi-nucleotide incorporation time courses, the EC syringe was prepared in a similar way but the pre-mixing concentration of TFIIS was held constant at 20 µM. GTP was added to the syringe containing ATP so that the pre-mixing concentrations of GTP and ATP were both 2 mM. Again, a 2-fold dilution occurs upon 1:1 mixing, and the listed reaction concentrations reflect this.

Gel electrophoresis and quantification of the products for both the single nucleotide and multi-nucleotide incorporation time courses were carried out as previously published [36].

### Elongation complex stability

EC stability experiments were carried out as previously published [19, 25]. EC solution was prepared as previously described. TFIIS (20 µM) was added to this EC solution and allowed to incubate for 10 min. This was then mixed 1:1 via pipetting with a solution containing 20 µM RNase A (Fisher Scientific) and 1.5 M KCl, yielding final concentrations of 10 µM and 750 mM, respectively. Time points at 25°C were taken continuously by removing 5 µl of the reaction mix and quenching with 25 mM DTT to inactivate RNase A. Reaction times varied from 8 s to 72 h, and products were separated and quantified as previously published [36].

### Data analysis

A previously published MATLAB toolbox called MENOTR [38] was used to fit quench-flow time courses. MENOTR uses a hybrid genetic algorithm (GA) and nonlinear least squares (NLLS) method to optimize parameters. Time courses were individually fit, kinetic parameters were optimized, and the average and standard deviation of at least three independent experiments are reported.

## Results

### Pol II cleavage enhancement saturates at micromolar TFIIS

To investigate the effect of TFIIS on Pol II-catalyzed nucleotide incorporation and cleavage, we used recombinant TFIIS (see Materials and methods) and Pol II purified from endogenous expression in *S. cerevisiae* as previously described [36]. We first sought to answer two questions: (i) When should TFIIS be added to Pol II? and (ii) What concentration of TFIIS is required to saturate Pol II-catalyzed cleavage?

Manual mixing single nucleotide incorporation experiments were performed to address these questions. For these experiments, two solutions were prepared: EC solution and ATP solution (see Materials and methods). Briefly, the EC solution contains a pre-assembled Pol II EC, illustrated in Fig. 1A. The EC contains Pol II bound to a radiolabeled 10-mer RNA, and the 10-mer serves as the starting point for any subsequent reactions. The ATP solution contains ATP, Mg^2+^, and heparin. ATP is the substrate for the next nucleotide incorporation, and Mg^2+^ is a necessary cofactor for this reaction. Heparin serves as a trap for any Pol II that may dissociate but does not trap TFIIS, as discussed below. Thus, these experiments are single turnover with respect to the Pol II EC but not TFIIS.

After preparation of the EC and ATP solutions, TFIIS was added to one of the solutions. Addition of TFIIS to the EC solution allows for TFIIS to pre-bind Pol II ECs before mixing with the ATP solution, i.e. “pre-mix TFIIS.” When TFIIS is added only to the ATP solution, TFIIS cannot encounter Pol II ECs until after mixing, i.e. “post-mix TFIIS.” For both the pre- and post-mix TFIIS conditions, enough TFIIS was added so that the [TFIIS] after mixing of the EC and ATP solutions ranged from 7 nM to 10 µM. In comparison, the final concentration of the Pol II EC is ∼40 nM after these solutions are mixed.

To initiate the single nucleotide incorporation and cleavage reactions, the EC and ATP solutions were mixed 1:1 via pipetting. Each reaction was allowed to proceed for 60 s at 25°C before quenching with 1 M HCl. Because the 10-mer RNA is radiolabeled, only product RNA 11-mer that contains the ^32^P-CMP is expected to be detected when the reactions are run on a denaturing gel. The reaction products are as follows: the starting 10-mer, the 11-mer single nucleotide incorporation product, and the CA dimer cleavage product detected only when TFIIS is added. The observation of the CA dimer cleavage product after nucleotide addition indicates that TFIIS bound to the EC, since Pol II will not catalyze significant endonucleolytic cleavage of the 3′ end of the RNA without TFIIS [25, 27, 30].

Each of these 60 s reactions collected at different total [TFIIS] were run on a denaturing polyacrylamide gel to separate products from reactants, and a representative gel of a pre-mix experiment is shown in Fig. 1B. The first lane represents the collection of a 60 s time point with no added TFIIS or ATP. As expected, only the radiolabeled 10-mer is present. The next lane represents a 60 s reaction with only ATP added and no TFIIS. In this lane, the starting 10-mer is no longer seen but extension products, 11- and 12-mer, are detected. As we have previously discussed, the 12-mer product can be due to misincorporation of a second AMP. An alternative explanation could be that low levels of contaminating GTP may be present in the ATP stock, resulting in a second incorporation event since the next cognate incorporation is GMP, yet no GTP was purposefully added [25, 29]. Also, because no TFIIS is added, there is no cleavage product detected, i.e. no CA dimer. On the other hand, 11-mer, 12-mer, and CA dimer formation are observed in the subsequent lanes where TFIIS, at increasing concentrations, is included. Further inspection of the gel reveals that as [TFIIS] increases, 11-mer and 12-mer signal decreases while CA dimer formation increases.

CA dimer formation was quantified using Eq. 1 as previously published [36]. The fraction of CA dimer was then plotted as a function of [TFIIS] to generate the isotherms shown in Fig. 1C. The average and standard deviation of three individual replicates are shown for both the pre-mix (blue dots in Fig. 1C) and post-mix (red dots in Fig. 1C) conditions. For both conditions, CA dimer formation saturates at [TFIIS] in the µM range, but the shapes of the isotherms deviate at pre-saturating concentrations. Also note that both isotherms saturate in less than two log units suggesting cooperativity.


(1) \begin{eqnarray*}
fra{c}_i\left( t \right) = \frac{{\frac{{\left[ {RN{A}_i} \right]\left( t \right)}}{{\mathop \sum \nolimits_i \left[ {RN{A}_i} \right]\left( t \right)}} - \frac{{\left[ {RN{A}_i} \right]\left( 0 \right)}}{{\mathop \sum \nolimits_i \left[ {RN{A}_i} \right]\left( 0 \right)}}}}{{1 - \frac{{\left[ {RN{A}_i} \right]\left( 0 \right)}}{{\mathop \sum \nolimits_i \left[ {RN{A}_i} \right]\left( 0 \right)}}}}
\end{eqnarray*}

To further interrogate the differences between the two approaches, the isotherms were subjected to NLLS analysis using the cooperative model in Eq. 2. The best fit of the pre-mix isotherm (blue line in Fig. 1C, Pre-Mix TFIIS in Table 1) yields a Hill coefficient *n* = 2.8 ± 0.9 and a *K*_D_ = (220 ± 30) nM. The best fit of the post-mix isotherm (red line in Fig. 1C, Post-Mix TFIIS in Table 1) yields a Hill coefficient *n* = 1.3 ± 0.4 and a *K*_D_ = (170 ± 50) nM.


(2) \begin{eqnarray*}
fra{c}_{CA} = \alpha \times \frac{{{{\left[ {\textit{TFIIS}} \right]}}^n}}{{{K}_D^n + {{\left[ {\textit{TFIIS}} \right]}}^n}}
\end{eqnarray*}

**Table 1.** TFIIS activity isotherm fit parameter values

Parameter	Pre-Mix TFIIS	Post-Mix TFIIS
α	0.84 ± 0.04	0.80 ± 0.06
*n*	2.8 ± 0.9	1.3 ± 0.4
*K*_D_ (nM)	220 ± 30	170 ± 50

Optimized parameter values and standard deviation for the fits shown in Fig. 1C. Standard deviation of the optimized parameter values is shown.

The simplest interpretation of the apparent cooperativity is that more than one TFIIS binds to the EC. To our knowledge, neither cooperativity nor any evidence of multiple TFIIS molecules binding to the EC has been reported. However, the observed cooperativity does not rule out multiple TFIIS binding events. That is, one TFIIS molecule can associate and dissociate from the EC multiple times during the reaction since these experiments are not single turnover with respect to TFIIS but are single turnover with respect to the EC.

In the post-mix experiment the TFIIS is mixed with the ATP and a large excess of heparin before addition to the EC to initiate the reaction. In this post-mix experiment we detect CA dimer. Consequently, it is clear that the heparin does not trap the TFIIS because if it did then no TFIIS would bind the EC after mixing. Thus, both the pre-mix and post-mix strategy are single turnover with respect to the EC but not single turnover with respect to TFIIS binding. That is to say, the experimental observable is not only reporting on the TFIIS that was bound at time zero in the pre-mix experiment. Thus, other explanations for the apparent cooperativity beyond two TFIIS protomers binding may explain the observation. To further test this, mechanistic path information is required, which the isotherms in Fig. 1C do not inherently provide.

### TFIIS binds after nucleotide incorporation

**Figure 2.**
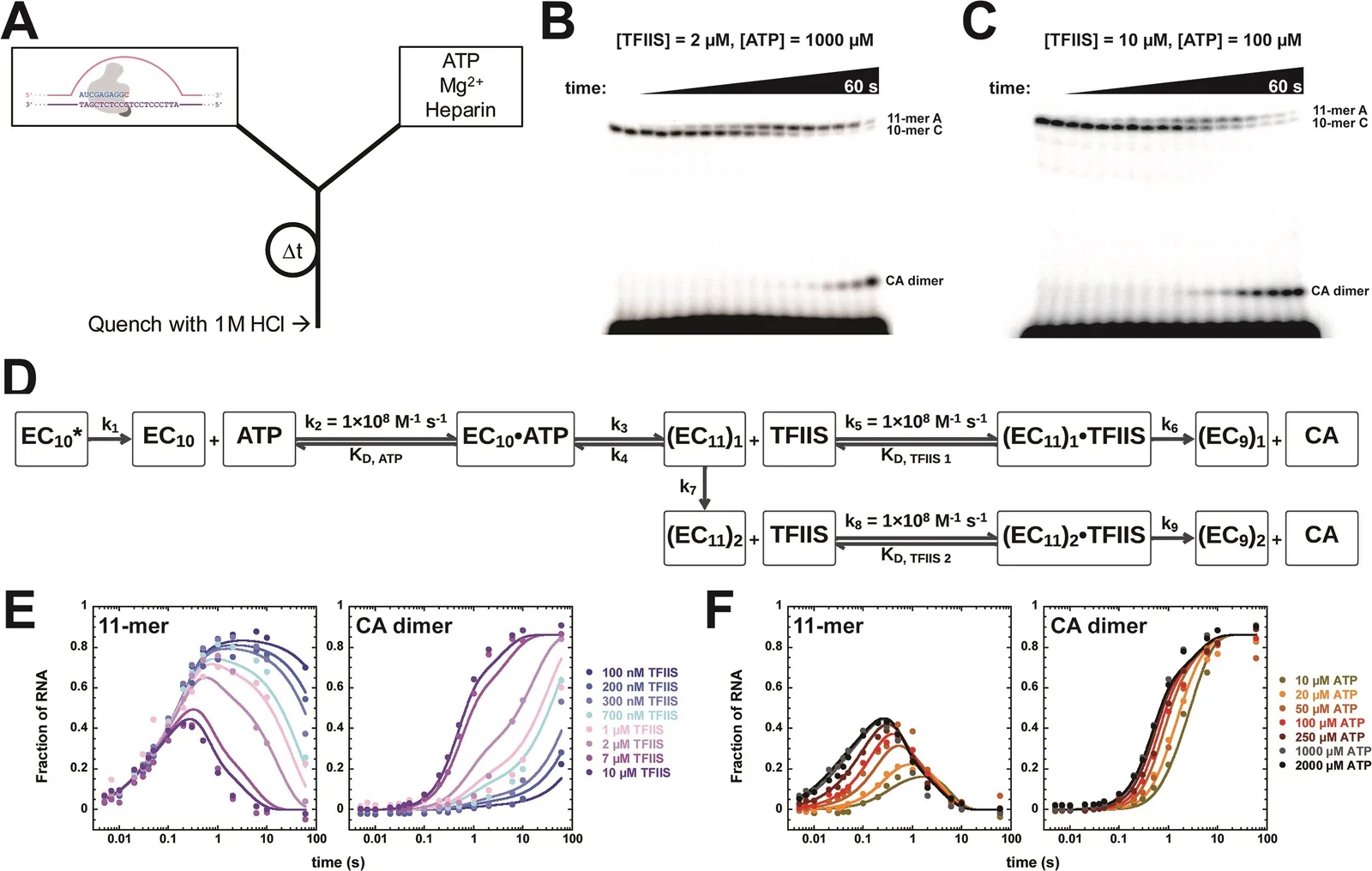
Single nucleotide incorporation kinetics. (**A**) Chemical quench-flow schematic. Radiolabeled ECs with TFIIS were loaded into one syringe of the chemical quench-flow, while a mixture of ATP, Mg^2+^, and heparin was loaded into the other syringe. These syringes were rapidly mixed and allowed to react for a user-defined period of time, Δ*t*, before being quenched with 1 M HCl. For varying [TFIIS] time courses, final [TFIIS] ranged from 100 nM to 10 µM while final [ATP] was 1000 µM. For varying [ATP] time courses, final [TFIIS] was 10 µM while final [ATP] ranged from 10 µM to 2000 µM. (**B**) Representative gel of the varying [TFIIS] time courses. Denaturing gel of a time course where final [TFIIS] = 2 µM and final [ATP] = 1000 µM. Each row represents a different intermediate or product while each column represents a different reaction time, Δ*t*. (**C**) Representative gel of the varying [ATP] time courses. Denaturing gel of a time course where final [TFIIS] = 10 µM and final [ATP] = 100 µM. Each row represents a different intermediate or product while each column represents a different reaction time, Δ*t*. (**D**) Single nucleotide incorporation scheme. Kinetic mechanism used to globally fit [TFIIS] and [ATP] dependencies simultaneously. (**E**) Representative fit of the [TFIIS] dependence. (**F**) Representative fit of the [ATP] dependence.

To acquire mechanistic information, we collected full time courses describing single nucleotide addition catalyzed by Pol II in the presence of TFIIS. Figure 2A shows a schematic of the experimental setup. Briefly, radiolabeled Pol II ECs were assembled with TFIIS as previously described (see Materials and methods). The assembled TFIIS-EC was then loaded into one syringe of the chemical quench-flow. The other syringe was loaded with a mixture of ATP, Mg^2+^, and heparin. The contents of the two syringes were rapidly mixed within 2 ms by the quench-flow and allowed to react for a user-defined period of time, Δ*t* = 5 ms – 60 s, before the reaction was rapidly quenched with 1 M HCl.

With this setup, two different concentration dependencies were tested. The first varied the final [TFIIS] from 100 nM to 10 µM while the final [ATP] was constant at 1 mM. The second held the final [TFIIS] constant at 10 µM while the final [ATP] ranged from 10 µM to 2 mM. Representative denaturing gel images for single nucleotide incorporation experiments carried out as a function of [TFIIS] or [ATP] are shown in Fig. 2B and C, respectively. Both gels show that, as the reaction time increases, the starting 10-mer disappears to form the 11-mer, and the 11-mer is cleaved to form the CA dimer.

Following quantification and normalization of the 11-mer and CA dimer signal, a kinetic mechanism that simultaneously describes both concentration dependencies was sought. To accomplish this, we started from the mechanism that best described single nucleotide addition catalyzed by Pol II [27]. However, since no cleavage activity was present in the absence of TFIIS the mechanism was modified as follows. The simplest modification that can be made assumes that, before ATP binding and incorporation, there are a fraction of ECs that are not bound and a fraction that are bound by TFIIS with no dissociation of this bound TFIIS (Supplementary Fig. S1A). Global fitting using this scheme (Supplementary Fig. S1B and C) resulted in the lowest goodness of fit, as indicated by the highest chi-squared, *Χ*^2^ = 6.23, of all schemes tested (see Supplementary Table S1).

The next model that was tested also had the assumption that the sample contained a fraction of ECs bound by TFIIS before ATP binding and incorporation, but a step was added to allow TFIIS to associate with or dissociate from ECs (Supplementary Fig. S1D). This scheme better described the data (Supplementary Fig. S1E and F), resulting in a decreased *Χ*^2^ = 1.30 (see Supplementary Table S1). However, some of the resulting elementary rate constants from the global optimization were faster than the detection limit of the quench-flow, which is an indication that they are not required in the analysis.

The schemes tested above both assumed TFIIS binding before ATP binding and incorporation. Thus, two schemes were written that only allowed for TFIIS binding after ATP binding and incorporation (Supplementary Fig. S1G and J). The resulting global fits using these models (Supplementary Fig. S1H and I, and K and L) described the experimental data to varying degrees, with the scheme in Supplementary Fig. S1G having an increased *Χ*^2^ = 2.64 compared to that of Supplementary Fig. S1D while Supplementary Fig. S1J showed a more comparable *Χ*^2^ = 1.64 (see Supplementary Table S1).

Considering the previously tested models, we arrived at a scheme that best described the time courses, resulting in the lowest observed *Χ*^2^ = 0.79 (see Supplementary Table S1) of all schemes tested. This reaction scheme is shown in Fig. 2D and includes a nucleotide binding step (*k*_2_ and *K*_D, ATP_), a reversible nucleotide incorporation step (*k*_3_ and *k*_4_), and two different TFIIS binding steps after nucleotide incorporation (*k*_5_, *K*_D, TFIIS 1_, *k*_8_, and *K*_D, TFIIS 2_). Although there are two distinct TFIIS binding steps in the scheme, the scheme allows for only a single TFIIS to be bound to a single Pol II EC. Figure 2E and F show a representative global fit for the [TFIIS] and [ATP] dependencies, respectively. Table 2 summarizes the optimized kinetic parameters of this global fit.

**Table 2.** Single nucleotide incorporation global fit

Parameter	Optimized value
*k*_1_ (s^-1^)	5.2 ± 0.1
*k*_2_ (M^-1^ s^-1^)	1 × 10^8^
*K*_D, ATP_ (µM)	520 ± 70
*k*_3_ (s^-1^)	65 ± 8
*k*_4_ (s^-1^)	4.2 ± 0.8
*k*_5_ (M^-1^ s^-1^)	1 × 10^8^
*K*_D, TFIIS 1_ (µM)	47 ± 5
*k*_6_ (s^-1^)	14.94 ± 0.05
*k*_7_ (s^-1^)	1.5 ± 0.3
*k*_8_ (M^-1^ s^-1^)	1 × 10^8^
*K*_D, TFIIS 2_ (µM)	450 ± 30
*k*_9_ (s^-1^)	13 ± 1
[EC_10_]/[EC_10, total_]	0.283 ± 0.008

Optimized values for parameters shown in Fig. 2D. The average and standard deviation of three individual fits are shown.

The scheme in Fig. 2D simultaneously describes both the TFIIS and ATP concentration dependent time courses. Moreover, the proposed mechanism indicates that, even though TFIIS is pre-incubated with Pol II ECs in one syringe of the chemical quench-flow, TFIIS does not bind the EC until after nucleotide addition. This predicts that if the TFIIS is included with the ATP, Mg^2+^, and heparin, followed by rapid mixing with the EC, i.e. post-mix TFIIS, then the kinetics of the reaction should be the same as when the TFIIS is included with the EC, i.e. pre-mix TFIIS.

**Figure 3.**
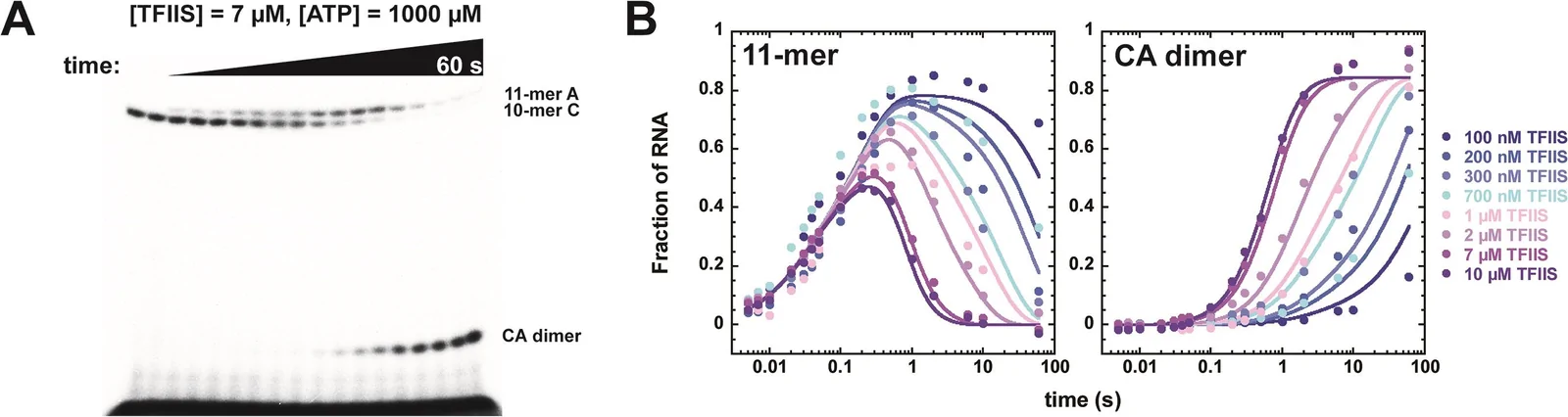
TFIIS post-mix time courses. (**A**) Representative gel of the post-mix time courses. Denaturing gel of a time course where final [TFIIS] = 7 µM and final [ATP] = 1000 µM. Each row represents a different intermediate or product while each column represents a different reaction time, Δ*t*. (**B**) TFIIS post-mix quench-flow time courses. Time courses were collected at varying final [TFIIS] and final [ATP] = 1000 µM. The global fit using the scheme in Fig. 2D is shown.

To test the prediction that TFIIS does not bind the EC until after nucleotide addition, we collected full single nucleotide addition “post-mix” time courses as a function of TFIIS concentration. The syringes were prepared in a similar way as the pre-mix time courses, but TFIIS was removed from the EC syringe and instead added to the syringe with ATP. A representative gel of these time courses is shown in Fig. 3A. The single nucleotide incorporation scheme in Fig. 2D was used to fit the post-mix quench-flow time courses in Fig. 3B and again well describes the data, resulting in a *Χ*^2^ = 1.30 (see Supplementary Table S1). While this goodness of fit value is increased compared to the global fit shown in Fig. 2E and F, we attribute this to the post-mix time courses not including the [ATP] dependence, thus lowering constraint on the ATP-dependent parameters, i.e. *K*_D,ATP_.

Fit parameter values of the TFIIS post-mix experiments are summarized in Table 3. The consistency of the parameters between the pre-mix and post-mix experimental approaches suggests that TFIIS does not appreciably bind the EC until after nucleotide incorporation. Moreover, given that formation of CA dimer is produced in the post-mix experiment, this is a clear indication that TFIIS does not bind heparin to any sufficient level to disrupt TFIIS binding to the EC. Again, this is evidence that these experiments are not single turnover with respect to TFIIS or ATP but are single turnover with respect to the EC.

**Table 3.** TFIIS post-mix fit parameter values

Parameter	Optimized value
*k*_1_ (s^-1^)	5.1 (2.7, 7.4)
*k*_2_ (M^-1^ s^-1^)	1 × 10^8^
*K*_D, ATP_ (µM)	410 (220, 590)
*k*_3_ (s^-1^)	52 (29, 76)
*k*_4_ (s^-1^)	2.9 (1.6, 4.2)
*k*_5_ (M^-1^ s^-1^)	1 × 10^8^
*K*_D, TFIIS 1_ (µM)	24 (13, 34)
*k*_6_ (s^-1^)	6.48 (3.53, 9.43)
*k*_7_ (s^-1^)	0.4 (0.2, 0.5)
*k*_8_ (M^-1^ s^-1^)	1 × 10^8^
*K*_D, TFIIS 2_ (µM)	50 (30, 80)
*k*_9_ (s^-1^)	5 (2, 7)
[EC_10_]/[EC_10, total_]	0.385 (0.230, 0.560)

**Figure 4.**
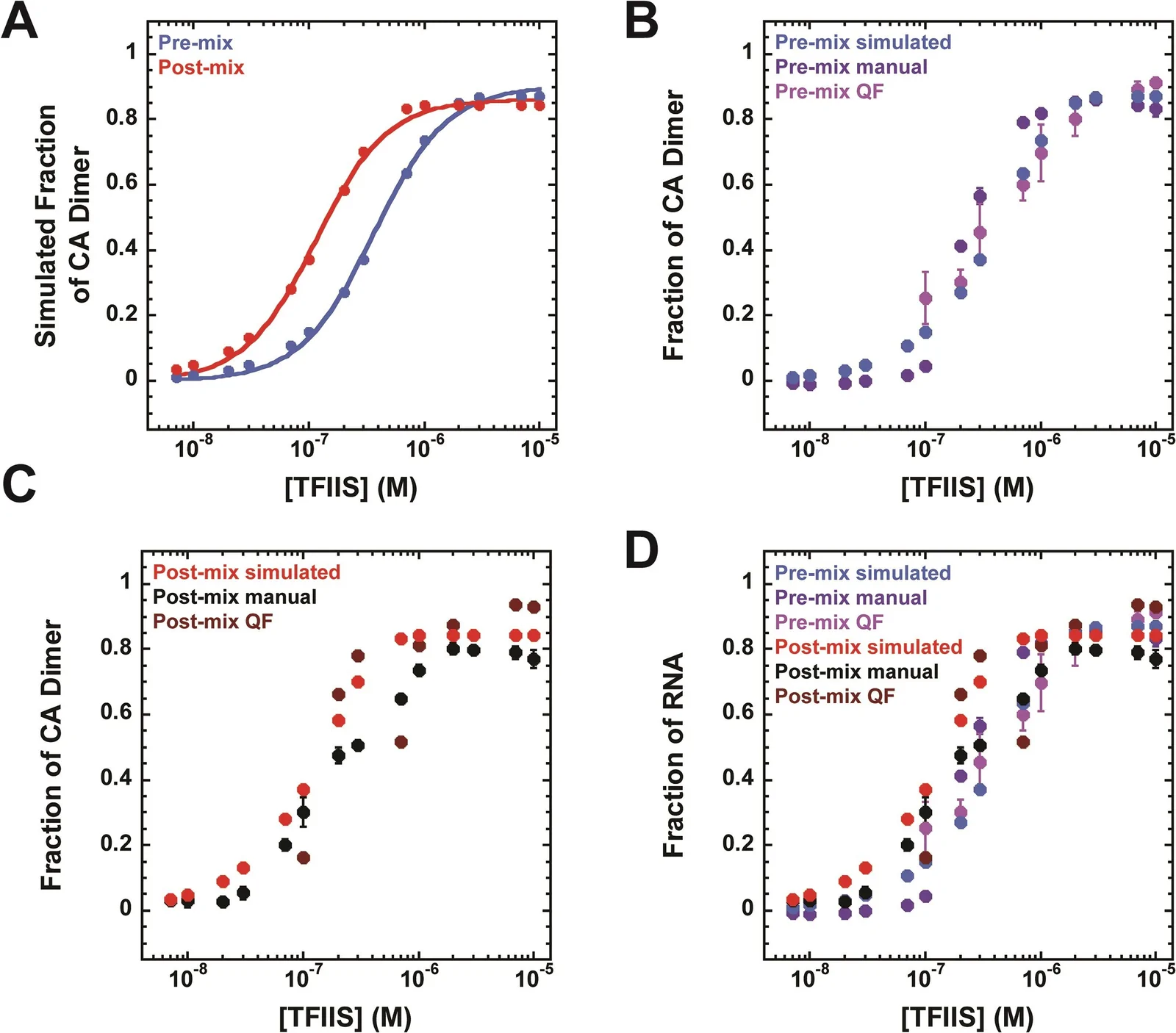
Comparison of simulated and experimental CA dimer formation. (**A**) Simulated activity isotherm from the single nucleotide incorporation scheme. Fraction of CA dimer formed at 60 s simulated from the kinetic mechanism in Fig. 2D plotted as a function of [TFIIS]. Simulations using the pre-mix fit parameters (see Table 2) and post-mix fit parameters (see Table 3) are shown in blue and red, respectively. Both are fit to a cooperative model (see Eq. 2). For the pre-mix simulation (blue), *n* = 1.4 ± 0.2 and *K*_D_ = (360 ± 60) nM. For the post-mix simulation (red), *n* = 1.4 ± 0.3 and *K*_D_ = (110 ± 20) nM. (**B**) Comparison of pre-mix CA dimer formation. CA dimer simulated using the pre-mix fit parameters (blue) overlaid with the fraction of CA dimer from pre-mix manual mixing experiments (purple) and the fraction of CA dimer at 60 s from pre-mix quench-flow experiments (magenta). (**C**) Comparison of post-mix CA dimer formation. CA dimer simulated using the post-mix fit parameters (red) overlaid with the fraction of CA dimer from post-mix manual mixing experiments (black) and the fraction of CA dimer at 60 s from a post-mix quench-flow experiment (dark red). (**D**) Comparison of pre- and post-mix CA dimer formation. Pre-mix CA dimer formation overlaid with post-mix CA dimer formation for all simulations and experiments.

Optimized values for the fit shown in Fig. 4B. Lower and upper bounds of the 68% confidence interval are shown.

The classical interpretation of cooperative binding is that multiple ligands are binding. Thus, it is tempting to interpret the apparent cooperative dependence of TFIIS binding to the EC as reflecting multiple TFIIS binding events. However, the result of the post-mix experiment in Fig. 3B indicates that these experiments are not single turnover with respect to TFIIS since the heparin is not trapping TFIIS when pre-incubated with a large excess of heparin. Thus, the final collected time point is not only reporting on what was bound at time zero but on the TFIIS that can bind the EC after mixing, i.e. before mixing with ATP and trap for free polymerase. Thus, the question emerged: does the model in Fig. 2D predict a cooperative dependence of CA dimer formation on [TFIIS] when the TFIIS can exchange with the EC after mixing?

We performed simulations using the Scheme in Fig. 2D to further test the prediction that a single site binding model with two isoforms can yield an apparent cooperative dependence on [TFIIS]. Specifically, we tested whether an activity isotherm simulated from the scheme in Fig. 2D could exhibit the apparent cooperativity observed in Fig. 1C. To this end, CA dimer formation after 60 s at varying [TFIIS] was simulated from the scheme shown in Fig. 2D. For the simulated pre-mix isotherm (Fig. 4A, blue), optimized parameters from the pre-mix time courses in Table 2 were used in the simulations. Similarly, for the simulated post-mix isotherm (Fig. 4A, red), optimized parameters from the post-mix time courses in Table 3 were used in the simulations. Both simulated isotherms were well described by a cooperative model (Eq. 2) with Hill coefficients of ∼1.4. For the pre-mix condition, this differs from the experimentally determined Hill coefficient of ∼2.8 which we hypothesize indicates that some TFIIS is pre-bound in a non-productive state to the EC in the pre-mix condition but must dissociate and rebind in a productive state, resulting in a higher degree of observed cooperativity.

These simulated data were then compared to experimental CA dimer formation at 60 s. Figure 4B compares the pre-mix 60 s CA dimer formation from the simulation, manual mixing, and chemical quench-flow. Figure 4C compares the post-mix 60 s CA dimer formation from the simulation, manual mixing, and chemical quench-flow. Together, these comparisons show that CA dimer formation simulated from the single nucleotide incorporation mechanism is similar to experimentally determined CA dimer formation. Importantly, simulations from a single site binding model with two different isoforms will produce isotherms that exhibit apparent cooperativity.

From the simulations and comparison to experimental results, we hypothesize that this is because TFIIS does not bind appreciably to ECs until after nucleotide incorporation which leads to similar formation of CA dimer regardless of when TFIIS is incubated with the EC. Overlaying of the pre- and post-mix CA dimer formation further supports this idea and can be seen in Fig. 4D.

### TFIIS has no effect on multiple nucleotide incorporations

We have previously reported that both single and multiple nucleotide incorporations are slower if A12.2 (TFIIS homolog) is removed from Pol I [18, 26]. Moreover, we found multi-nucleotide addition to be reversible for Pol I ΔA12 but irreversible for wild-type Pol I. Similarly, Pol II, which naturally lacks TFIIS, was found to exhibit reversible multi-nucleotide additions like Pol I ΔA12 [30]. Thus, we asked the question: does adding TFIIS to Pol II make multi-nucleotide additions both faster and irreversible, i.e. more “Pol I like”? Further, since the single nucleotide addition experiments with TFIIS imply that TFIIS binds after nucleotide addition, we asked the question: will TFIIS remain bound to an actively transcribing Pol II?

**Figure 5.**
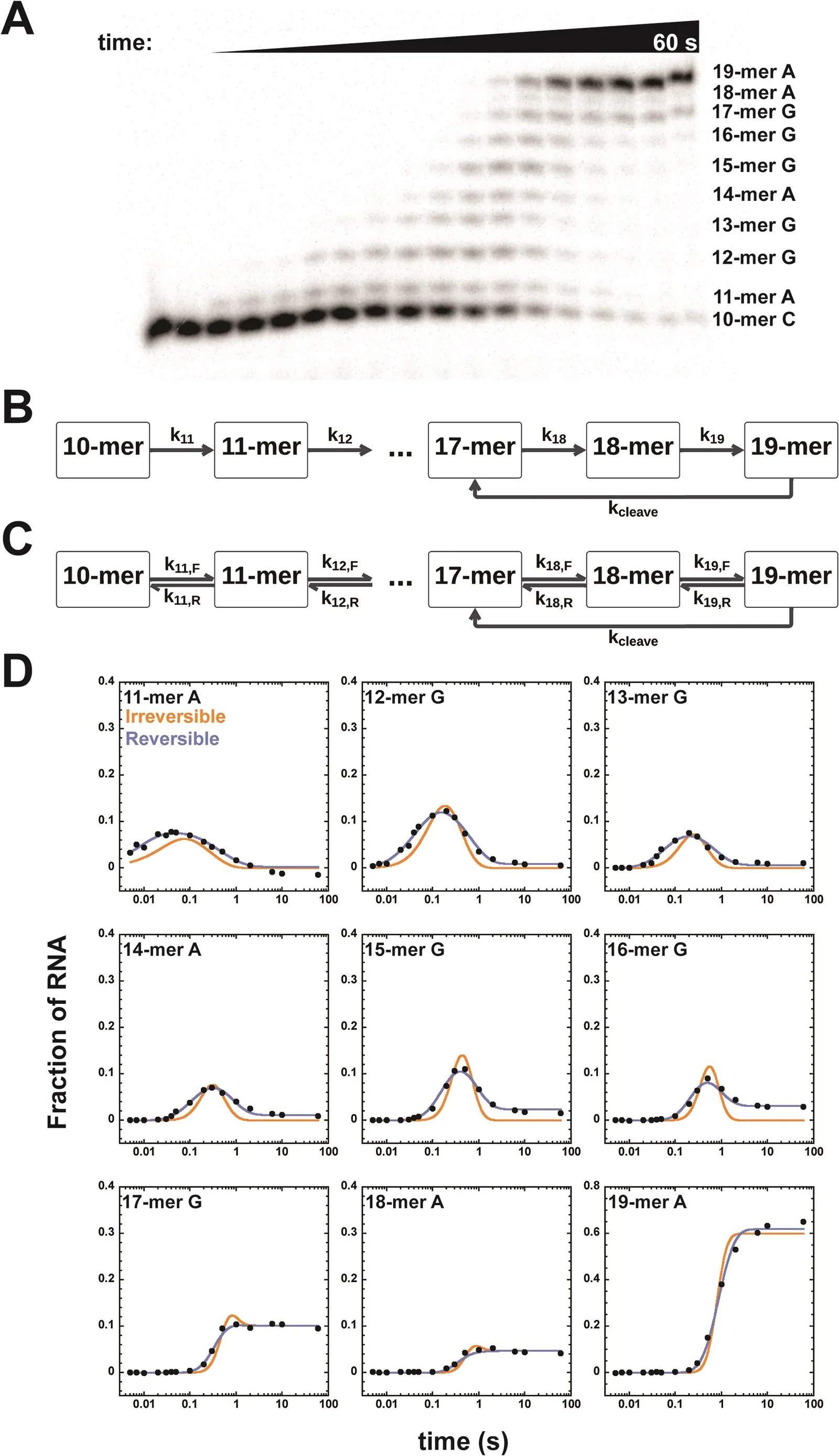
Multi-nucleotide incorporation kinetics. (**A**) Representative multi-nucleotide incorporation gel. Denaturing gel of a time course where final [TFIIS] = 10 µM and final [ATP] = [GTP] 1000 µM. Each row represents a different intermediate or product while each column represents a different reaction time, Δ*t*. (**B**) Irreversible multi-nucleotide incorporation scheme. (**C**) Reversible multi-nucleotide incorporation scheme. (**D**) Representative fit of the multi-nucleotide incorporation data. Global fits across all nine intermediates or products to the irreversible scheme (orange line) and reversible scheme (blue line) are shown.

To test the effect of TFIIS on multiple nucleotide incorporations GTP was added to the ATP, Mg^2+^, and heparin syringe shown in Fig. 2A. This modification allows for the collection of time courses for nine successive nucleotide incorporations. In this experiment a saturating [TFIIS] of 10 µM was used while the final [ATP] and [GTP] concentrations were 1000 µM. Reaction time, Δ*t*, was again varied from 5 ms to 60 s. A representative gel of Pol II-catalyzed multi-nucleotide incorporation in the presence of TFIIS is shown in Fig. 5A. As Δ*t* increases, disappearance of the starting 10-mer, formation and disappearance of intermediates, and accumulation of the final 19-mer product can be observed. Quantification and normalization of each intermediate and the final product were carried out as described [36].

Two reaction schemes were written to determine a kinetic mechanism that describes the multi-nucleotide incorporation time courses. The first scheme, shown in Fig. 5B, has irreversible nucleotide incorporation steps and a single endonucleolytic cleavage step after the final 19-mer product is produced. The second scheme shown in Fig. 5C introduces reversibility at each nucleotide incorporation step but still has a single cleavage step for the 19-mer. Both schemes were used to globally fit across all nine reaction intermediates and products, and representative fits for the irreversible and reversible schemes are shown in Fig. 5D.

The irreversible scheme (orange line in Fig. 5D) is unable to describe the persistence of several intermediates, resulting in a *Χ*^2^ = 0.015 (see Supplementary Table S1). On the other hand, the reversible scheme (blue line in Fig. 5D) does account for how these intermediates persist in time and gives rise to an improved *Χ*^2^ = 0.006. Optimized kinetic parameters from global fitting using the reversible scheme are summarized in Table 4. The forward and reverse rate constants have average values (33 ± 19) s^-1^ and (31 ± 27) s^-1^, respectively, with no apparent correlation between observed rate constant and nucleotide position (see Supplementary Fig. S2).

**Table 4.** Multi-nucleotide incorporation global fit

Parameter	Optimized value
*k*_11, F_ (s^-1^)	13 ± 3
*k*_11, R_ (s^-1^)	80 ± 30
*k*_12, F_ (s^-1^)	68 ± 6
*k*_12, R_ (s^-1^)	22 ± 5
*k*_13, F_ (s^-1^)	44 ± 4
*k*_13, R_ (s^-1^)	58 ± 7
*k*_14, F_ (s^-1^)	50 ± 10
*k*_14, R_ (s^-1^)	34 ± 9
*k*_15, F_ (s^-1^)	44 ± 7
*k*_15, R_ (s^-1^)	21 ± 5
*k*_16, F_ (s^-1^)	17 ± 3
*k*_16, R_ (s^-1^)	13 ± 5
*k*_17, F_ (s^-1^)	13 ± 1
*k*_17, R_ (s^-1^)	3.9 ± 0.5
*k*_18, F_ (s^-1^)	33 ± 5
*k*_18, R_ (s^-1^)	50
*k*_19, F_ (s^-1^)	19 ± 6
*k*_19, R_ (s^-1^)	0.6 ± 0.5
*k*_cleave_ (s^-1^)	1.1 ± 0.3

Optimized values for parameters shown in Fig. 5C. The average and standard deviation of three individual fits are shown.

The reverse of a nucleotide incorporation reaction is the pyrophosphorolysis reaction. Simply, incorporation of the next cognate nucleotide results in production of RNA of length *n +* 1 and pyrophosphate. If this pyrophosphate is not released from the active site of the polymerase, it can be used in the pyrophosphorloysis reaction to produce RNA of length *n* and the original substrate NTP. Thus, the observation of reversibility indicates that pyrophosphate is slow to release.

The scheme that best describes Pol II-catalyzed multi-nucleotide incorporation in the presence of TFIIS has reversibility at each step. This is identical to what we reported for Pol II catalyzed multi-nucleotide addition in the absence of TFIIS [30]. This leaves us with two possibilities. Either TFIIS is bound to the EC for each incorporation but does not impact each nucleotide addition or TFIIS does not bind until the 19-mer is formed and no additional incorporations are possible. To differentiate between these models, a post-mix multi-nucleotide incorporation time course was collected where TFIIS was mixed with ECs at the same time as ATP and GTP, and this time course overlays well with the time courses collected with TFIIS pre-mixed with ECs (see Supplementary Fig. S3). From these data, we conclude that TFIIS binds after the last incorporation which is consistent with the single nucleotide addition results reported here.

### Pol II elongation complex stability is unaffected by the presence of TFIIS

We have previously shown that the A12.2 subunit of Pol I is an intrinsic destabilizer of the Pol I EC [19]. Thus, we hypothesize that TFIIS may destabilize the Pol II EC. Moreover, if TFIIS is bound to the EC before nucleotide addition, then this would predict an impact on the stability of the EC. Therefore, to further test if TFIIS is bound to the EC or if it binds after nucleotide addition, we performed an RNase protection assay that reports on the stability of the EC [19, 25].

**Figure 6.**
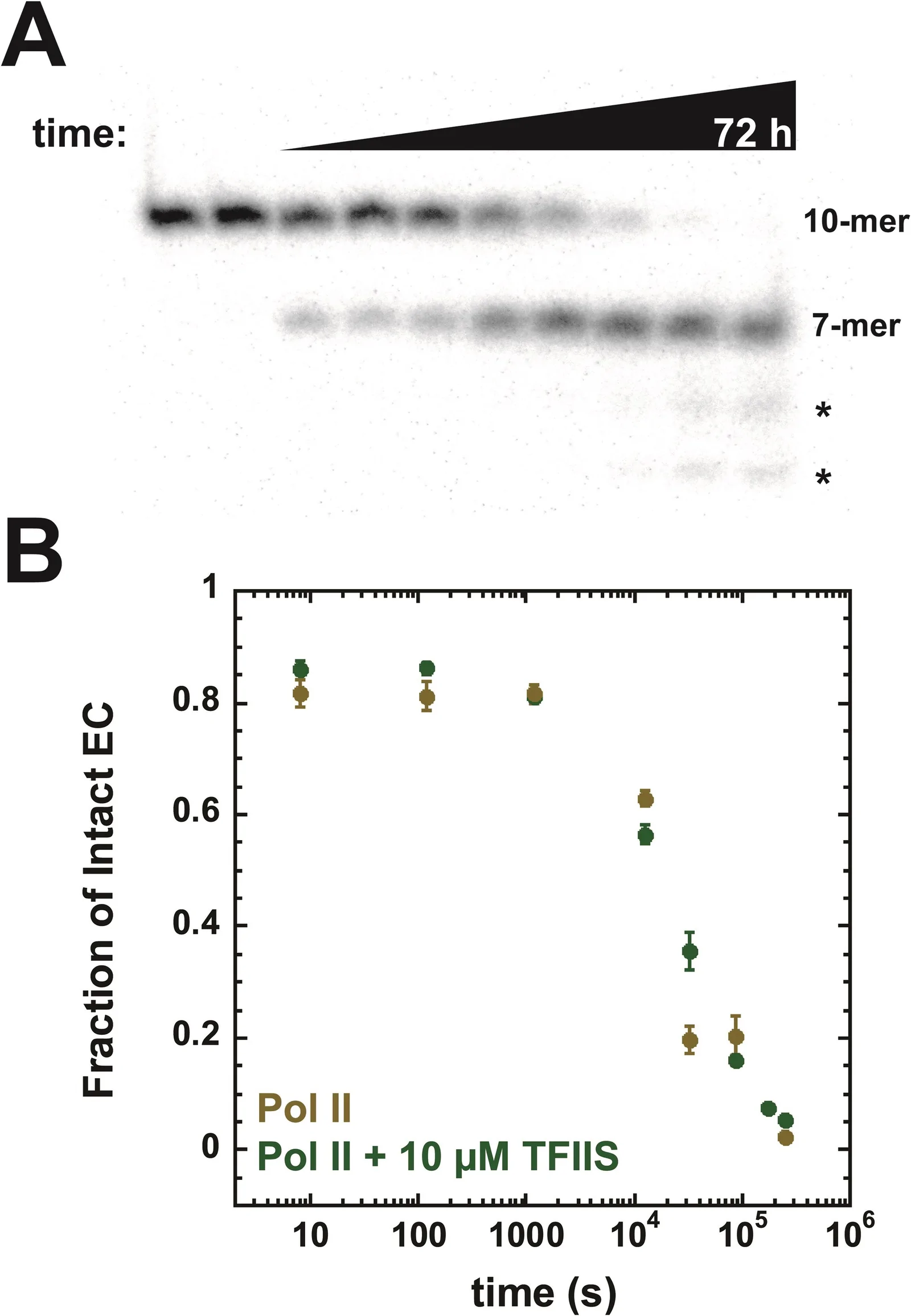
EC stability. (**A**) Representative EC stability gel. Denaturing gel of EC stability experiment where each row represents a different intermediate or product while each column represents a different reaction time, Δ*t*. The * indicates smaller RNA species that were not included in quantification and normalization of the 10-mer and 7-mer. (**B**) Pol II EC stability comparison. Fraction of intact ECs for Pol II (gold) and Pol II with 10 µM TFIIS (green) plotted as a function of time. For both conditions, the average and standard deviation of three replicates is shown.

Briefly, Pol II ECs are radiolabeled and assembled in the presence of TFIIS. These ECs are then mixed with 750 mM KCl and RNase A. The high concentration of KCl destabilizes electrostatic interactions between the polymerase and the nucleic acids, and RNase A will cleave RNA that has dissociated from an EC. A representative denaturing gel of this experiment can be seen in Fig. 6A. As the time of incubation with 750 mM KCl increases, the signal of starting 10-mer disappears and 7-mer formation following RNase A cleavage is observed.

Pol II EC stability in the presence and absence TFIIS is compared in Fig. 6B. As seen in the plot, the fraction of intact Pol II ECs decay identically for Pol II alone and Pol II in the presence of 10 µM TFIIS. We hypothesize two possible explanations for this similarity in EC stability. First, TFIIS is bound to Pol II ECs but does not affect the overall stability of the EC. Second, TFIIS is not bound to Pol II ECs that are not actively undergoing transcription elongation and would thus have no effect on EC stability as measured by this assay. Along with our observations from the nucleotide incorporation experiments reported above, we favor the second hypothesis of TFIIS not being bound to the Pol II EC.

## Discussion

In contrast to the single bacterial RNAP, eukaryotes have evolved three unique RNAPs. In eukaryotes, a clear division of labor has emerged where Pol I, II, and III are responsible for rRNA, mRNA, and tRNA synthesis, respectively. The structural differences between the three Pols have been well documented [2, 5, 10–15]. However, the impact of the structural divergence on function has not been fully described. To fill this knowledge gap, we have focused on the mechanistic differences that may have evolutionarily emerged and led to three Pols with unique transcription responsibilities.

One obvious difference between the three Pols are the subunits or transcription factors responsible for conferring endonuclease activity. Robust endonuclease activity allows for recovery of stalled transcription ECs and is proposed to aid in transcription fidelity through proofreading [32, 33, 39]. For the eukaryotic Pols, A12.2 and C11 confer this activity to Pol I and Pol III, respectively, and are intrinsic subunits of their respective polymerases [2, 7, 8]. Conversely, robust endonuclease activity for Pol II is acquired through the recruitment of a trans-acting factor, TFIIS [7, 8, 39].

Removal of the A12.2 subunit from Pol I also results in the loss of subunits A49 and A34.5 [40, 41]. These two subunits are homologous to the transcription factors TFIIF and TFIIE in Pol II. Thus, Pol I in the absence of these three subunits is, structurally, more Pol II like. Thus, we asked the question, if we remove the A12.2 subunit would the transcription mechanism catalyzed by Pol I ΔA12 be more Pol II like?

As expected, removal of the A12.2 subunit from Pol I did result in a loss of endonuclease activity. Moreover, Pol I did catalyze slower single and multi-nucleotide addition reactions like Pol II [18, 26]. But the most unexpected finding was that the multi-nucleotide addition reactions catalyzed by Pol I ΔA12 and Pol II both exhibited reversibility in each repeating round of multi-nucleotide addition [26]. Since the reverse of a nucleotide addition reaction is pyrophosphorolysis, this indicates that pyrophosphate must be slow to release in the absence of A12.2 and TFIIS in Pol I and Pol II, respectively.

Slow pyrophosphate release was further tested by adding pyrophosphate to the multi-nucleotide addition reactions catalyzed by Pol I, Pol I ΔA12, and Pol II [26, 30]. Consistent with slow pyrophosphate release, Pol I ΔA12 and Pol II exhibited minimal pyrophosphate sensitivity. On the other hand, the time courses collected with Pol I in the presence of pyrophosphate required a model with reversible bond formation. Recall, Pol I in the absence of pyrophosphate is best described by repeating cycles of irreversible steps implying that pyrophosphate is fast to release in Pol I.

Our previous observations inspired the work reported here, with the next obvious question being: can we make Pol II more Pol I like by adding TFIIS to the single and multi-nucleotide addition reactions? Although adding TFIIS to these reactions did confer robust endonuclease activity, it did not appear to impact the elementary rate constants describing nucleotide addition. If there is an impact on the nucleotide addition step, the presence of TFIIS may have slowed down the single nucleotide addition reaction, i.e. ∼80 s^-1^ [27] and ∼65 s^-1^ (*k*_3_ in Table 2) for Pol II and Pol II with TFIIS, respectively. Moreover, from the post-mix experiment we measure a rate constant of ∼52 s^-1^ (*k*_3_ in Table 3). However, all these differences are within experimental error.

Upon addition of TFIIS, we do observe the expected enhancement in Pol II endonuclease activity. Surprisingly, the endonuclease activity appeared to depend cooperatively on the [TFIIS]. However, to our knowledge, multiple TFIIS molecules binding to an EC has not been reported. In fact, previous studies are consistent with 1:1 binding of Pol II and TFIIS [42, 43]. Moreover, self-assembly of TFIIS monomers to form higher order oligomers has not been suggested nor examined in detail [44, 45], and we only observe monomeric TFIIS at the highest concentrations used in this study (see Supplementary Fig. S4).

Although the endonuclease activity appears to cooperatively depend on [TFIIS], it is important to note that those experiments are single turnover with respect to the EC but not with respect to TFIIS. Therefore, they do not reflect a thermodynamically rigorous TFIIS binding isotherm. For this to be the case, we would need to trap TFIIS after mixing with ATP and heparin. Heparin is clearly not trapping TFIIS or else the post-mixing experiment where TFIIS is incubated with ATP and heparin would not yield a result consistent with TFIIS binding to the EC, i.e. the observation of endonucleolytic cleavage products. We have yet to be able to successfully trap TFIIS, so, in the experiments reported here, TFIIS is able to freely exchange between solution and binding to the transcription EC.

Despite the limitations in interpreting the activity isotherm, the isotherm does clearly report on the amount of TFIIS required to saturate the activity and indicates that it’s in the micromolar concentration regime. This saturating [TFIIS] is higher than the final [Pol II] of ∼40 nM in our *in vitro* experiments and the [TFIIS] is at least in 25-fold excess. Previous investigations of protein abundance in *S. cerevisiae* suggest that the *in vivo* ratio of TFIIS to Pol II is much lower, approximately 1:2 [46, 47]. Pol II in yeast cells still have robust endonuclease activity despite the sub-saturating intracellular [TFIIS], and the local concentration in the nucleus is likely much higher to encourage cleavage enhancement.

The *in vitro* experiments reported here suggest that endonuclease activity is kinetically controlled. Our single nucleotide incorporation experiments and modeling (see Fig. 2, Table 2, Fig. 3, and Table 3) suggest that TFIIS binding is rate-limiting at sub-saturating concentrations. Assuming diffusion-controlled binding kinetics of TFIIS, the rate constant of TFIIS binding is 10 s^-1^ at our lowest tested concentration of TFIIS of 100 nM. This is slower than the ∼65 s^-1^ nucleotide addition step and on the same order of magnitude as the two cleavage steps: ∼15 and ∼13 s^-1^. As TFIIS concentration decreases, this difference in rate constants would become further apparent with TFIIS binding emerging as the slowest step.

Kinetic control may be important for transcriptional proofreading and/or pause recovery. It has been previously reported that the subsequent nucleotide addition step following a misincorporation is five to twenty times slower than nucleotide addition following a correct incorporation [33]. This would decrease our reported nucleotide addition rate constant from ∼65 s^-1^ to a rate constant in the range of 3 to 13 s^-1^. With nucleotide addition slowed, TFIIS can bind and enhance endonuclease activity to remove the misincorporation before the next addition can occur. By the same argument, a pause can be recovered by yielding sufficient time for TFIIS to bind and catalyze cleavage. With the misincorporation removed, nucleotide addition can again proceed quickly, which no longer favors TFIIS binding. Thus, TFIIS-mediated proofreading of transcription elongation is kinetically controlled by TFIIS binding.

In this way, TFIIS differs from its homologous counterparts, A12.2 and C11, as they are bona fide subunits of Pol I and Pol III, respectively. Thus, endonuclease activity of these polymerases is not limited by binding since they are always present in the EC. However, based on the relative rate constants for incorporation vs. cleavage there is a kinetic competition in Pols I and III. For a single nucleotide incorporation catalyzed by Pol I, the rate constant of nucleotide addition is ∼200 s^-1^ while the subsequent cleavage rate constant is ∼0.4 s^-1^ [16]. Pol III has a nucleotide addition rate constant of ∼60 s^-1^ and two cleavage rate constants of ∼1.4 and ∼0.005 s^-1^ [25]. Thus, for these Pols, transcription can proceed at a much faster rate since the subunit is always present to cleave when needed. If a misincorporation, leading to a stall, occurs then the rate constant for incorporation following a misincorporation is likely sufficiently slow that cleavage can occur. However, in Pol I and III the cleavage reaction is not limited by a bimolecular binding step but only a unimolecular cleavage step. Thus, Pol II has an additional level of regulation of cleavage through both the bimolecular binding step, which is regulated by the cellular TFIIS concentration, as well as the unimolecular cleavage step which must be of comparable order of magnitude to the incorporation following a misincorporation.

We have also reported that A12.2 not only influences Pol I cleavage but also affects nucleotide incorporation. In the context of a single nucleotide incorporation, Pol I bond formation is irreversible and fast, ∼200 s^-1^ [16]. Pol I lacking A12.2 (ΔA12 Pol I) differs as the bond formation step is reversible and slower, ∼60 s^-1^ [18]. The observation of reversibility requires that pyrophosphate remains bound in the active site for a sufficient amount of time to allow for reversal of the bond formation step since no additional pyrophosphate was provided. From these observations, we concluded that A12.2 is involved in each nucleotide addition and does more than just confer endonuclease activity, but it accelerates pyrophosphate release.

Due to the homology between A12.2 and TFIIS, we hypothesized that addition of TFIIS to Pol II would result in Pol II being more “Pol I like.” However, our results show that this hypothesis is not correct. For a single nucleotide incorporation, Pol II bond formation is reversible and slower than Pol I at ∼80 s^-1^ versus ∼200 s^-1^ [27]. Upon addition of TFIIS, we see Pol II bond formation is unchanged as it is still reversible and ∼65 s^-1^. However, even when TFIIS is pre-incubated with the EC, there is no evidence that it is bound to the EC before the nucleotide addition reaction occurs. Thus, whether TFIIS accelerates each nucleotide addition remains unknown. Unlike Pol I, our current evidence suggests that TFIIS is not involved in the Pol II bond formation step for a single nucleotide addition.

On the other hand, making Pol II more Pol I like may not only require the A12.2 homolog, TFIIS, but may also require the addition of TFIIF and TFIIE. Those transcription factors are homologous to the A49 and A34.5 subunits in Pol I and the C37 and C53 subunits in Pol III. The A49 and A34.5 subunits dissociate from Pol I upon removal of the A12.2 subunit [40, 41]. Interestingly, Pol III exhibits a similar phenomenon. If the A12.2/TFIIS homolog, C11, is removed, C37 and C53 also dissociate from Pol III [48]. Thus, the endonuclease homologues appear to serve as platforms for the association of other subunits/transcription factors.

The multi-nucleotide addition experiments are also consistent with TFIIS not forming a stable complex with the EC through multiple additions. Pol II multi-nucleotide addition is best described by reversible steps with an average value of (33 ± 15) s^-1^ [30], and Pol II with TFIIS also exhibits reversible steps with an average value of (33 ± 19) s^-1^. A difference between Pol II and Pol II with TFIIS is not seen until the final nucleotide incorporation at position 19. After this addition Pol II stops incorporating since the next cognate nucleotide, U, is not present. In the presence of TFIIS, Pol II catalyzes endonucleolytic removal of the last two nucleotides to form a 17-mer and nucleotide addition begins again. This is consistent with TFIIS binding after nucleotide addition and TFIIS not binding until the EC stalls.

In sum, our findings further support mechanistic divergence between Pol II and Pols I/III that impacts how endonucleolytic cleavage is carried out. Compared to Pol I and Pol III, regulation of cleavage in Pol II is first controlled at the cellular level by the free concentration of TFIIS and second by the kinetic competition between cleavage and incorporation. In contrast, Pol I and Pol III contain endonucleolytic cleavage activity that is regulated only through the kinetic competition between incorporation and cleavage. However, the story may be more complex as there remains a need to determine the impact of TFIIF and TFIIE in transcription along with TFIIS.

## Supplementary Material

gkag874_Supplemental_Files

## Data Availability

Data and associated analyses are available via Zenodo at https://doi.org/10.5281/zenodo.21998508.
