# Supplementary material for: TFIIS enhancement of Pol II cleavage is under kinetic control
Source: Nucleic Acids Res. 2026 Sep 16;54(17):gkag874. doi: 10.1093/nar/gkag874 (PMC13578497; doi:10.1093/nar/gkag874)
Supplement: Supplementary file 1 [file gkag874_supplemental_files.zip › Requijo Pol II TFIIS supplementary - revised.docx]

# **SUPPLEMENTARY DATA**

**Supplementary Figure S1. Single Nucleotide Incorporation Test Schemes.** (A) Test scheme 1. (B) Representative fit of the [TFIIS] dependence for test scheme 1. (C) Representative fit of the [ATP] dependence for test scheme 1. (D) Test scheme 2. (E) Representative fit of the [TFIIS] dependence for test scheme 2. (F) Representative fit of the [ATP] dependence for test scheme 2. (G) Test scheme 3. (H) Representative fit of the [TFIIS] dependence for test scheme 3. (I) Representative fit of the [ATP] dependence for test scheme 3. (J) Test scheme 4. (K) Representative fit of the [TFIIS] dependence for test scheme 4. (L) Representative fit of the [ATP] dependence for test scheme 4.

| **Table S1. Chi-Squared Goodness of Fit for Each Scheme** | |
| --- | --- |
| **Single Nucleotide Incorporation Fits** | |
| **Scheme** | **Χ^2^** |
| Fig. S1 A – Pre-Mix TFIIS | 6.23 |
| Fig. S1 D – Pre-Mix TFIIS | 1.30 |
| Fig. S1 G – Pre-Mix TFIIS | 2.64 |
| Fig. S1 J – Pre-Mix TFIIS | 1.64 |
| Fig. 2 D – Pre-Mix TFIIS | 0.79 |
| Fig. 2 D – Post-Mix TFIIS | 1.30 |
| **Multi-Nucleotide Incorporation Fits** | |
| Fig. 5 B – Pre-Mix TFIIS | 0.015 |
| Fig. 5 C – Pre-Mix TFIIS | 0.006 |

**Supplementary Figure S2. Observed Rate Constant vs. Nucleotide Position for Multi-nucleotide Incorporation.** Forward (k_obs,F_, grey) and reverse (k_obs,R_, red) rate constants from **Table 4** plotted as a function of nucleotide position. The average and standard deviation of k_obs,F_ across all nucleotide positions is (33 ± 19) s^-1^ while the average and standard deviation of k_obs,R_ is (31 ± 27) s^-1^.

**Supplementary Figure S3. Comparison of Pre-Mix and Post-Mix Multi-Nucleotide Incorporation.** Individual plots of each intermediate and product for pre-mix (grey) and post-mix (red) multi-nucleotide incorporation time courses. Time courses for both conditions were collected at final [TFIIS] = 10 µM and final [ATP] = [GTP] 1000 µM. For the pre-mix condition, the average and standard deviation of three replicates is shown.

**Supplementary Figure S4. TFIIS Analysis.** (A) Raw mass spectrometry data of recombinant TFIIS. (B) Processed mass spectrometry data of TFIIS showing a single peak at ~34.9 kDa. (C) c(s) distribution from sedimentation velocity AUC experiments conducted at 7 and 10 µM TFIIS.
